# Association between early ventricular arrhythmias and mortality in destination vs. bridge patients on continuous flow LVAD support

**DOI:** 10.1038/s41598-021-98109-2

**Published:** 2021-09-28

**Authors:** Zeina Jedeon, Rebecca Cogswell, Jessica Schultz, Lisa Von Wald, Ranjit John, Henri Roukoz

**Affiliations:** 1 Division of Cardiology, Department of Medicine, University of Minnesota, Minneapolis, MN USA; 2 Division of Cardiothoracic Surgery, Department of Surgery, University of Minnesota, Minneapolis, MN USA; 3 Electrophysiology Section, Division of Cardiovascular Medicine, University of Minnesota, 420 Delaware Street SE, MMC 508, Minneapolis, MN 55455 USA

**Keywords:** Cardiac device therapy, Arrhythmias, Heart failure

## Abstract

The association between ventricular arrhythmias (VAs) and mortality in patients supported by continuous flow left ventricular assist devices (LVAD) remains controversial. To evaluate the association between pre-implantation, early (≤ 30 day) post-implantation VAs and mortality in bridge to transplant (BTT) and destination therapy (DT) LVAD patients, separately. The risk factors for post LVAD VAs were also investigated. In this observational cohort study, we included 341 patients who received a first time, continuous flow LVAD between January 1st 2010 and July 30th 2018. We used Kaplan–Meier curves and multivariable cox regression analyses to study the association between VAs and mortality in the BTT and DT populations. The mean age of the cohort was 58 ± 14 years, with 82% males, 53% had ischemic cardiomyopathy, and 45% were DT. The mean follow-up was 2.2 ± 2.1 years. In both BTT and DT cohorts, pre LVAD VAs were not associated with mortality after LVAD implantation (log-rank p = 0.95 and p = 0.089, respectively). In the BTT population, early post-LVAD VAs were not statistically associated with increased mortality (log rank p = 0.072). In the DT patients, early post LVAD VAs were associated with a 67% increase in the hazards rate of mortality on LVAD support (HR 1.67 [1.05–2.65], p = 0.029). The final model was adjusted for type of cardiomyopathy, INTERMACS profile, glomerular filtration rate, post LVAD atrial fibrillation, age and cerebrovascular events. Early post-LVAD VA is common after LVAD implantation and is an independent predictor of mortality in the DT LVAD population.

## Introduction

Continuous-flow left ventricular assist device (LVAD) implantation improves survival and quality of life in patients with end-stage heart failure^1^. Although less likely to be acutely life-threatening, ventricular arrhythmias (VAs) are common after LVAD implantation. The association between VAs and mortality in patients with continuous flow LVAD remains controversial. Since the UNOS allocation system change in October of 2018 in the United States, newly implanted LVAD patients are more likely to be destination therapy (DT), and the existing bridge to transplant (BTT) patients are facing longer times on LVAD support^2^. Collectively this raises the importance of understanding the risk of preexisting VAs and the clinical significance of post LVAD Vas in both patient populations. Furthermore, patients implanted as DT are usually older and have more comorbidities compared to patients listed for transplantation, hence could be more sensitive to VAs. The purpose of the present analysis was to test the association between pre-implantation and early post-implantation early (≤ 30 days) VAs and mortality in patients on continuous flow LVAD support in the DT and BTT populations, separately. The risk factors for post LVAD VAs were also investigated.

## Methods

### Patient population

This observational cohort study was conducted with approval from the Institutional Review Board of the University of Minnesota. All research was performed in accordance with relevant guidelines and regulations and in accordance with the Declaration of Helsinki. Informed consent was waived for the University of Minnesota Mechanical Support Database and not required for retrospective data entry as approved by the Institutional Review Board of the University of Minnesota. The study included 344 consecutive patients who had received LVADs as either DT or BTT between January 1st 2010 and July 30th 2018 at the University of Minnesota Medical Center, Minneapolis, MN. Patients with an LVAD designated as bridge to recovery were excluded (n = 3).

### The University of Minnesota Mechanical Support Database

The following demographic and clinical covariate data are included in the LVAD database which is maintained though data extraction and manual chart review. The database includes all patients who do not opt out of research undergoing LVAD implantation at the University of Minnesota. The data collected includes type of cardiomyopathy, LVAD pump type, Interagency Registry for Mechanically Assisted Circulatory Support (INTERMACS) profile, glomerular filtration rate, and presence of absence of diabetes, hyperlipidemia, history of coronary artery bypass graft (CABG) surgery, chronic obstructive pulmonary disease, transient ischemic attack or stroke, peripheral vascular disease, presence or absence implantable cardioverter defibrillator (ICD) and history of atrial fibrillation (AF). Echocardiographic parameters including pre-LVAD left ventricular ejection fraction (EF), pre-LVAD left ventricular end diastolic dimension (LVEDD), and pre-LVAD left ventricular end systolic dimension (LVESD) were also collected.

### Definitions and outcomes

Arrhythmia device data was retrospectively extracted from the device clinic database and from chart review. Sustained ventricular arrhythmias were defined as either a VA ≥ 30 s, causing hemodynamic instability or treated with cardioversion either externally or with an implantable cardioverter defibrillator (ICD). Early post-LVAD VA was defined as occurring less than or equal to the first 30 postoperative days. New onset early post LVAD VA was defined as Early post-LVAD VA without history of pre-LVAD VA. Late post LVAD VA as defined as occurring beyond 30 days after LVAD implantation. Follow-up started at the day of LVAD implantation and ended with either the date of the heart transplant, the patient’s death or the last day of data entry (October 15th, 2019).

The study population was divided into BTT and DT populations. DT patients were designated as such if they had a permanent contra-indication to transplant (such as age) and if DT was designated in the chart at the time of LVAD implantation. If the patient was not on the transplant list at the time of LVAD implantation but the intention was cardiac transplant and the patient was later listed, the patient was designated BTT for the purposes of this study.

### Statistical analysis

Continuous variables were evaluated for normality and are shown as mean ± SD or medians as appropriate. Categorical variables are presented as percentages. Categorical variables were analyzed using Fisher’s exact and/or Chi-square tests. Continuous variables were analyzed using non-parametric (Kruskal–Wallis) or student’s t-test as appropriate. We performed multivariate logistic regression models for non-time-dependent outcomes and adjusted for factors significant in the univariate analysis. Kaplan–Meier Curves and proportional hazards models were used to assess survival and time dependent outcomes and the log-rank test was used to compare survival estimates.

For mortality analysis, patients who underwent heart transplantation were censored at the time of cardiac transplantation. Potential confounders of the relationship between ventricular arrhythmias and post-LVAD mortality including age, race, sex, INTERMACS profile, cardiomyopathy type, prior sternotomy, diabetes mellitus, were forced in the models and tested for significance in a univariate exploratory analysis. These were then incorporated in a forward and backward stepwise fashion using the likelihood ratio test for significance to arrive at a final adjusted model^2^. A p-value < 0.05 was considered statistically significant. All statistical analyses were performed using the JMP Pro 15.0 (SAS Institute Inc, Cary, NC).

### Disclosures

Dr Henri Roukoz has received consulting fees from Boston Scientific and speaking fees from Medtronic. Dr Rebecca Cogswell has the following disclosures: Abbott Lab: consultant, speaker’s bureau, Medtronic: Heart Failure Advisory Board, speaker’s bureau, husband’s employment, NuPulse: national principle investigator. Dr Ranjit John has the following disclosures: Abbott Lab—consultant, research grants, Medtronic: consultant, research grants.

## Results

### Baseline characteristics

The study cohort was comprised of 341 patients. The mean age of the cohort was 58 ± 14 years, 82% were male, 53% had ischemic cardiomyopathy, and 45% were designated as DT at the time of LVAD implantation. The INTERMACS profile was ≤ 2 in 65.5% of patients. The mean follow-up was 2.2 ± 2.1 years. Pre-LVAD VAs were present in 51% of the entire cohort and early post LVAD VAs occurred in 37% of patients, respectively. Baseline characteristics of the general cohort according to BTT or DT are presented in Table 1.

**Table 1:** Baseline characteristics of LVAD cohort.

	Overall cohort (n = 341)	DT group (n = 155)	BTT group (n = 186)	P value	Early VA (n = 128)	No early VA (n = 213)	P value
Age (years ± SD)	58.1 ± 14.1	64.4 ± 12.9	53.2 ± 12.8	** < 0.001**	60.3 ± 11.7	57.0 ± 15.1	**0.027**
Male gender n (%)	278 (81.5%)	125 (80.6%)	153 (82.3%)	0.76	104 (81.2%)	174 (81.7%)	0.92
Caucasian n (%)	270 (86.5%)	125 (88.0%)	145 (85.3%)	0.55	104 (88.9%)	166 (85.1%)	0.64
ICM n (%)	179 (52.5%)	79 (51.0%)	100 (53.8%)	0.65	75 (58.6%)	104 (48.8%)	0.08
Pre-LVAD VA n (%)	173 (51.3%)	71 (46.1%)	102 (54.8%)	0.051	80 (63.0%)	93 (43.7%)	** < 0.001**
**LVAD type**							
Heartmate II	234 (68.2%)	110 (71.9%)	124 (66.3%)	0.26	92 (71.9%)	142 (67.0%)	0.21
Heartmate III	57 (16.6%)	25 (16.3%)	30 (16.0%)		15 (11.7%)	40 (18.9%)	
HeartWare	52 (15.2%)	18 (11.8%)	33 (17.6%)		21 (16.4%)	30 (14.1%)	
INTERMACS ≤ 2 n (%)	222 (65.5%)	100 (64.9%)	122 (65.9%)	0.80	84 (65.6%)	138 (65.4%)	0.97
Mean BMI (n ± SD)	29.0 ± 5.8	28.5 ± 6.0	29.5 ± 5.6	0.01	30.0 ± 5.6	28.5 ± 5.8	**0.017**
CAD n (%)	251 (74.5%)	119 (76.8%)	132 (72.5%)	0.43	97 (77.0%)	154 (73.0%)	0.41
PAD n (%)	57 (17.0%)	36 (23.4%)	21 (11.5%)	**0.001**	14 (11.1%)	43 (20.5%)	**0.027**
CVA/TIA n (%)?	72 (21.1%)	31 (20.0%)	41 (22.0%)	0.23	44 (34.9%)	64 (30.5%)	0.40
MI n (%)	112 (33.2%)	51 (32.9%)	61 (33.5%)	0.84	41 (32.5%)	71 (33.6%)	0.83
CABG n (%)	54 (16.0%)	29 (18.7%)	25 (13.7%)	0.16	21 (16.7%)	33 (15.6%)	0.80
Prior sternotomy n (%)	130 (37.8%)	62 (40.5%)	65 (35.6%)	0.35	55 (42.6%)	75 (34.9%)	0.15
Prior circulatory support n (%)	48 (13.9%)	17 (11.1%)	31 (16.2%)	0.17	24 (18.6%)	24 (11.2%)	0.08
DM n (%)	146 (43.3%)	76 (49.0%)	70 (38.5%)	0.07	58 (46.0%)	88 (41.7%)	0.44
HTN n (%)	171 (50.1%)	78 (50.3%)	93 (51.1%)	0.84	68 (54.0%)	103 (48.8%)	0.36
HPL n (%)	132 (39.2%)	65 (41.9%)	67 (36.8%)	0.30	51 (40.5%)	81 (38.4%)	0.70
AF n (%)	134 (39.8%)	76 (49.0%)	58 (31.9%)	**0.001**	50 (39.7%)	84 (39.8%)	0.98
ICD n (%)	228 (67.7%)	107 (69.0%)	121 (66.5%)	0.65	92 (73.0%)	136 (64.5%)	0.10
COPD n (%)	70 (20.1%)	40 (25.8%)	30 (16.5%)	**0.043**	27 (21.4%)	43 (20.4%)	0.82
Mean GFR (n ± SD)	59.8 ± 20.7	56.4 ± 19.7	62.8 ± 21.1	**0.003**	56.5 ± 18.5	61.8 ± 21.7	**0.021**
Pre-EF (n ± SD)	19.9 ± 8.2	19.6 ± 8.3	20.1 ± 8.2	0.67	19.8 ± 7.8	19.8 ± 8.5	0.99
Pre-LVEDD (n ± SD)	7.0 ± 1.0	6.8 ± 1.1	7.2 ± 1.0	**0.006**	7.05 ± 1.0	6.95 ± 1.0	0.42
Pre-LVESD (n ± SD)	6.3 ± 1.1	6.1 ± 1.1	6.4 ± 1.1	**0.008**	6.32 ± 1.0	6.23 ± 1.1	0.50

*AF* atrial fibrillation, *BMI* body mass index, *BTT* bridge to transplant, *CABG* coronary artery bypass graft, *CAD* coronary artery disease, *COPD* chronic obstructive pulmonary disease, *CVA* cerebrovascular accident, *DM* diabetes mellitus, *DT* destination therapy, *GFR* glomerular filtration rate, *HPL* hyperlipidemia, *HTN* hypertension, *ICD* Implantable cardioverter-defibrillator, *ICM* ischemic cardiomyopathy, *INTERMACS* Interagency Registry for Mechanically Assisted Circulatory Support, *LVAD* left ventricular assist device, *LVEDD* left ventricular end-diastolic diameter, *LVESD* left ventricular end-systolic diameter, *MI* myocardial infarction, *PAD* peripheral artery disease, *TIA* transient ischemic attack, *VA* ventricular arrhythmia.

P values presented in bold are statistically significant (<0.05).

DT patients were more likely to have peripheral vascular disease and chronic obstructive pulmonary disease compared to BTT patients. In addition, DT patients had lower glomerular filtration rates and smaller left ventricular end diastolic dimension compared to BTT patients. Early VA post LVAD developed in 37.4% of the entire cohort, in 38.3% of the DT group and in 36.6% of the BTT group. The patients who had early VA were older, had more pre LVAD VA, a higher BMI and a lower GFR when compared to patients without early post LVAD VA (Table 1).

### Predictors of early and late VA

Pre-LVAD VA was the sole independent predictor of early post-LVAD VA (adjusted OR = 2.16 [1.33–3.51], p = 0.002) after adjusting for the predictors in the univariate analysis: age, type of cardiomyopathy, INTERMACS profile, peripheral vascular disease and GFR.

In the univariate analysis, both pre-LVAD VA (OR = 1.82 [1.17–2.83] p = 0.008) and early post-LVAD VA (OR = 1.87 [1.18–2.99] p = 0.008) were associated with increased late post-LVAD VA. In the multivariate analysis, early post-LVAD VA was not an independent risk factor for late post-LVAD VA (OR = 1.66 [0.99–2.76] p = 0.053), while pre-LVAD VA was still an independent risk factor (OR = 1.71 [1.05–2.77] p = 0.031) along with an INTERMACS ≤ 2 (OR = 1.75 [1.06–2.92] p = 0.031).

### Ventricular arrhythmias and mortality

The 3-year mortality during follow-up was 32.9% of the overall population, 41.9% of the DT population and 25.8% of the BTT population. There was a significantly higher 3-year mortality in patients with early post LVAD VA compared to patients with no early post LVAD VA (39.8% vs 29.1%, p = 0.041). Of note, there was no difference in 30 day mortality between patients with early post LVAD VA and patients with no early post LVAD VA. Patients with early post LVAD VA had a significantly higher 3 month mortality (20.2% vs 9.3%, p = 0.004) but no difference in mortality beyond the first 3 months after LVAD implantation (34% vs 33.1%, p = 0.76, and Log rank p = 0.96) compared to patients with no early post LVAD VA. A total of 74 (21.7%) patients received heart transplantation during follow-up, with 72 (38.7%) patients in the BTT group and 2 (1.3%) patients in the DT group. Mortality at the end of follow-up was 44.3% in the entire cohort, 34.4% in the BTT group and 57.2% in the DT group. The outcomes are presented in Table 2.

**Table 2:** Post-LVAD outcomes.

	Overall cohort (n = 341)	DT group (n = 153)	BTT group (n = 188)	P value	Early VA (n = 128)	No early VA (n = 213)	P value
Early post-LVAD VA n (%)	128 (37.4%)	59 (38.3%)	69 (36.6%)	0.58	–	–	–
New onset early post LVAD VA n (%)		25 (20.6%)	22 (15.6%)	0.29	47 (36.7%)	–	–
Late post-LVAD VA n (%)	174 (54.7%)	71 (50.0%)	103 (58.5%)	0.25	74 (64.3%)	100 (49.3%)	**0.009**
VA ablation n (%)	21 (6.2%)	8 (5.2%)	13 (7.0%)	0.74	16 (12.5%)	5 (2.3%)	** < 0.001**
30-day mortality	26 (7.6%)	16 (10.5%)	10 (5.2%)	0.07	14 (10.8%)	12 (5.6%)	0.08
90-day mortality	46 (13.4%)	30 (19.6%)	16 (8.4%)	**0.002**	26 (20.2%)	20 (9.3%)	**0.004**
Mortality beyond 90 days	97 (32.8%)	57 (46.3%)	40 (23.1%)	** < 0.001**	35 (34.0%)	62 (33.1%)	0.76
3-year mortality n (%)	113 (32.9%)	65 (41.9%)	48 (25.8%)	**0.002**	51 (39.8%)	62 (29.1%)	**0.041**
Heart transplant n (%)	74 (21.7%)	2 (1.3%)	72 (38.7%)	** < 0.001**	26 (20.3%)	48 (22.5%)	0.63
Death or heart transplant	217 (63.3%)	89 (57.4%)	128 (68.8%)	**0.005**	87 (68.0%)	130 (61.0%)	0.20
Death at end of follow-up	152 (44.6%)	87 (56.9%)	65 (34.6%)	** < 0.001**	63 (49.2%)	89 (41.8%)	0.18

P values presented in bold are statistically significant (<0.05).

*BTT* bridge to transplant, *DT* destination therapy, *LVAD* left ventricular assist device, *RV* right ventricle, *VA* ventricular arrhythmia.

**Figure 1:**
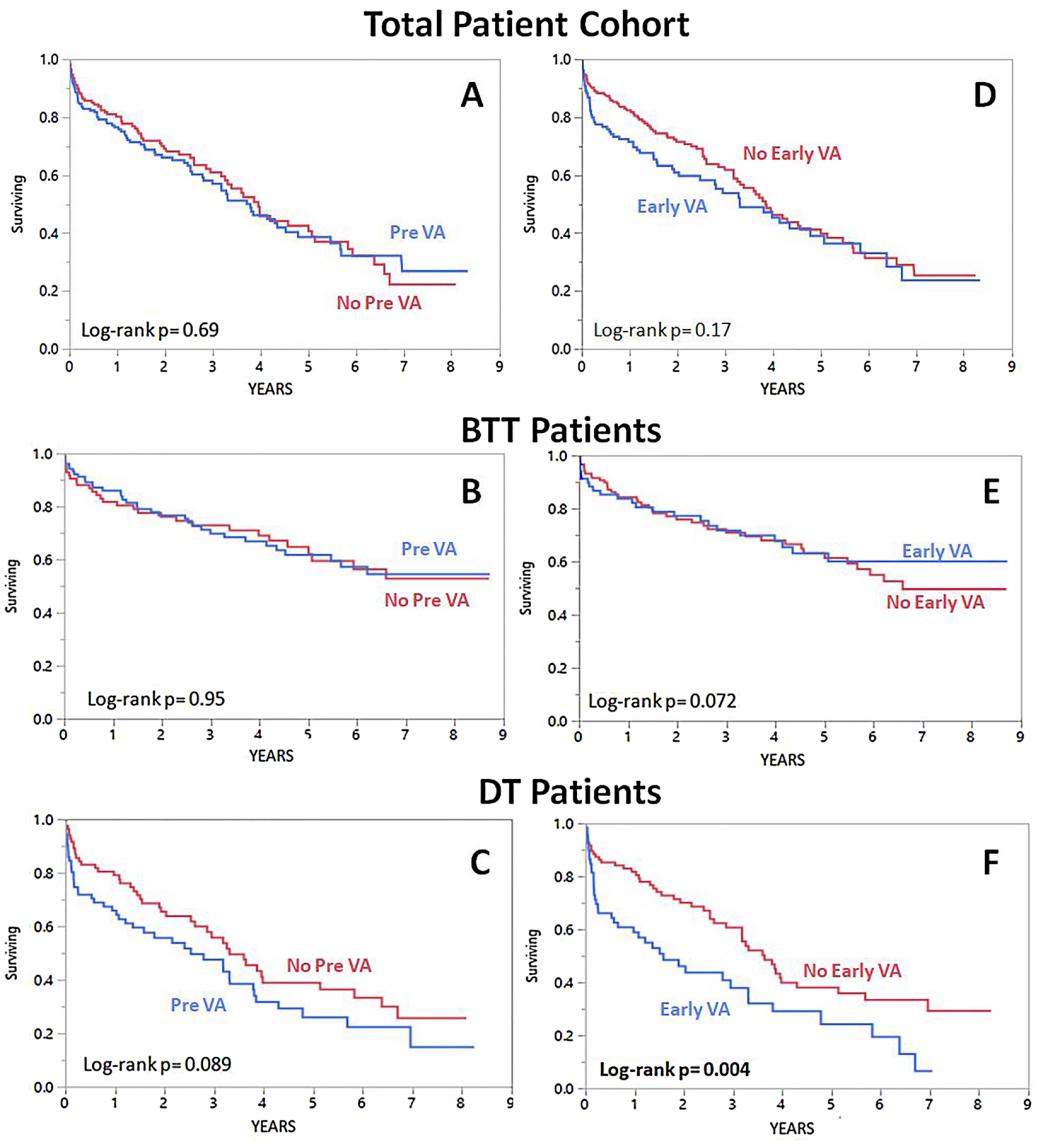
Kaplan–Meyer curves for mortality dichotomized by the existence of pre-LVAD VA (**A–C**) and early post-LVAD VA (**D–F**) in all the patient cohort, the BTT group and the DT group.

In the overall cohort, pre-LVAD VA and early post-LVAD VA were not associated with increased mortality (Fig. 1). Pre-LVAD VA were not associated with a higher mortality rate in the BTT and DT population (Log-rank p = 0.95 and p = 0.089, respectively). Early post-LVAD VA was not significantly associated with a higher mortality in the BTT population (Log-rank p = 0.072) but was associated with increased mortality in the DT population (Log-rank p = 0.004). In the final multivariate model, early post LVAD VA were associated with a 67% increase in the hazards rate of mortality after follow-up in the DT group (HR 1.67 [1.05–2.65], p = 0.029). The final model was adjusted for type of cardiomyopathy, INTERMACS profile, glomerular filtration rate, post LVAD AF, age, BMI and CVA (Table 3).

**Table 3:** Univariate and multivariate analysis for mortality in patients with DT LVAD.

Variable	Univariate analysis			Multivariate analysis		
	HR	[95% CI]	P value	HR	[95% CI]	P value
ICM	2.07	[1.34–3.20]	**0.001**	2.22	[1.35–3.65]	**0.002**
INTERMACS ≤ 2	1.75	[1.13–2.72]	**0.013**	2.23	[1.29–3.85]	**0.004**
GFR*	0.21	[0.09–0.50]	** < 0.001**	0.98	[0.97–0.99]	**0.019**
Early post VA	1.90	[1.24–2.91]	**0.003**	1.60	[1.02–2.53]	**0.041**
Post AF	1.60	[1.03–2.47]	**0.036**	1.52	[0.85–2.72]	0.15
Age	2.19	[0.79–6.73]	0.13	1.01	[0.98–1.04]	0.41
CVA	1.73	[1.07–2.80]	**0.025**	1.21	[0.74–1.98]	0.44
BMI	2.34	[0.96–5.42]	0.06	1.04	(0.98–1.09]	0.24

P values presented in bold are statistically significant (<0.05).

*Per unit change, *AF* atrial fibrillation, *CI* confidence interval, *CVA* cerebrovascular accident, *DT* destination therapy, *GFR* glomerular filtration rate, *HR* hazard ratio, *ICM* ischemic cardiomyopathy, *INTERMACS* Interagency Registry for Mechanically Assisted Circulatory Support, *LVAD* left ventricular assist device, *VA* ventricular arrhythmias.

When we studied the factors associated with short term mortality, early post LVAD VA was associated with a significant increase in 3 month mortality in the DT group (31.6% vs 12.5%, p = 0.004) but not in the BTT group (11.1% vs 6.7%, p = 0.29) compared to patients without early post LVAD VA. Mortality beyond the first 3 months was not significantly different between patients with early post LVAD VA and patients with no early post LVAD VA in neither BTT (Log rank p = 0.26) or DT (Log rank p = 0.09) groups.

**Figure 2:**
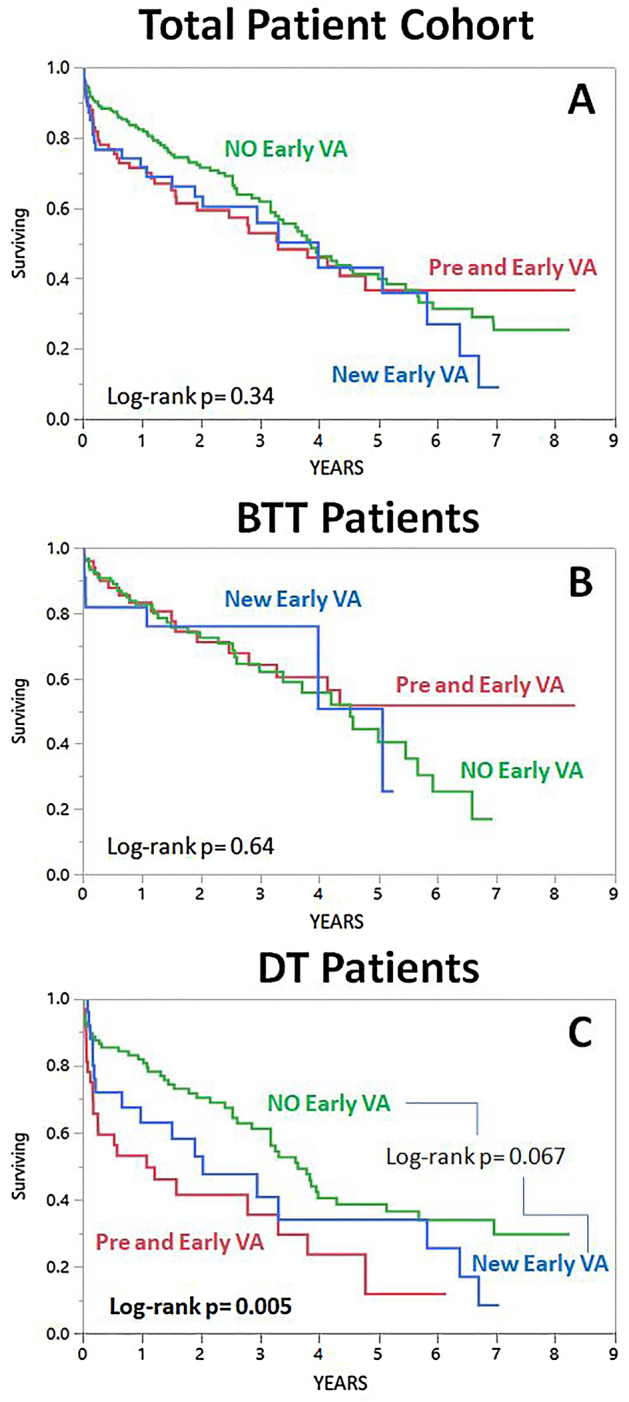
Kaplan-Meyer curves for mortality divided by the existence of new onset early post-LVAD VA (Early post LVAD VA without history of VA prior to LVAD implantation. in all the patient cohort (**A**), the BTT group (**B**) and the DT group (**C**).

About a third of patients (36.7%) with early post LVAD VA had new onset VA without prior history of pre-LVAD VA. New onset VA was not associated with increased mortality in the overall cohort and the BTT group. It was associated with increased mortality in the DT group but did not reach statistical significance (Log rank p = 0.067) when compared only to the patients with no early post LVAD VA (Fig. 2).

## Discussion

The principal findings of this analysis were: (1) that early post-LVAD VA is common even in the contemporary continuous flow LVAD era (38%), while late post-LVAD VA occurred in 55% during long term follow-up; (2) pre-LVAD VA but not early post-LVAD VA was an independent risk factor for late post-LVAD; and (3) early post-LVAD VA was associated with a higher mortality in the DT population, especially in the first few months after LVAD implantation.

Early VAs after LVAD implantation are common with a reported incidence between 13 and 25% in patients with continuous flow LVADs^3–6^. Our study reports a higher incidence of early VAs (37%). This may be driven by variation in the definition of early VAs, with some studies using the first week rather than the first 30 days after LVAD implantation^7^. This difference may also be explained by the high percentage of patients in our cohort with a history of VAs prior to LVAD (51%) relative to other studies using the same definition, suggesting a higher risk population. This reflects practice patterns prior to the UNOS allocation system change (Oct 18, 2018) where patients with refractory VAs and low ejection fraction were likely to receive LVADs as a BTT given the long wait times in UNOS Region 7.

Pre-LVAD VT was an independent predictor of both early and late post-LVAD VA. Pre-LVAD VA has been described as a predictor of post LVAD VA in general^6,8^, early and late post-LVAD VA in particular^3–5,9^. This suggests that pre-existing substrate may be a large driver for ventricular arrhythmias in patients after LVAD. Indeed, multiple studies reported that the majority of ventricular arrhythmias ablated in the LVAD population are related to scar remote form the LVAD canula^10–13^. While pre-LVAD was an independent predictor of late post LVAD VA, early post LVAD VA was not after adjusting for pre-LVAD VA. This suggests that having early VA does not significantly increase the chances of late VA beyond the pre-existing substrate. This result is different that the findings by Galand et al. where early post LVAD VAs were still an independent risk factor for late post LVAD VAs^9^. This difference is probably due to the fact that our population is higher risk with more pre-LVAD VAs (33.4% versus 51.3%) and more substrate to drive post LVAD VAs. Many patients without pre-existing VAs, however, develop VAs after LVAD implantation. In our study, about a third of patient who developed early post LVAD VA had new onset VA without prior history of VA before LVAD implantation, suggesting that there are still mechanisms that are specific to early post LVAD VAs including electrolyte abnormalities, suction events, inotropic agent use, perioperative ischemia and acute right ventricular dysfunction and LVAD cannula associated scar^4,7,14,15^.

The association of early post LVAD VA and mortality we observed in the DT cohort is consistent with other observational studies that have also demonstrated an increased mortality with VAs, particularly early post-LVAD VAs^4,5,7,16^. A recent meta-analysis of 9 observational studies including 1,179 patients also revealed an association between post-LVAD VAs and mortality^17^. However, this association is not consistent^18^. A recent analysis of data from the INTERMACS registry showed no such association^19^. It is known that INTERMACS reporting is often incomplete, which limits the strength of the conclusions drawn with using this data. This highlights the importance of high-quality data to answer clinically relevant questions in the LVAD population. Given the event rate in this population, a large sample size is often not needed to have confidence around a result.

In our study, early post LVAD VA was independently associated with mortality in the DT population, but not in the overall cohort or the BTT population. In the DT population, the increase in morality occurred early after LVAD implantation and the separation in survival curves persisted until the end of follow up. This is corroborated by the association between early post LVAD VA and the 3 month mortality but not with mortality beyond the first 3 months as observed in our study. Furthermore, new onset early post LVAD VA without prior history of VA was associated with increased mortality especially in the first few months after LVAD implantation. This association did not reach statistical significance likely because of a decrease in power driven by a smaller sample size. There was not a statistically significant increase in mortality in the BTT VAD recipients who developed early post-operative ventricular arrhythmias, however there was a trend in that direction with a log rank p value of 0.07. One possible explanation for the lack of separation in survival curves in the BTT cohort is the potential for early transplantation, however the practice at the University of Minnesota is to wait at least 3 months before re-listing BTT patients after surgery. Another potential mechanism for the association between early post LVAD VAs and early mortality is that VAs can lead to worsening right ventricular failure in the high risk post-operative period^3^. The most likely explanation is that patients in the DT population have more co-morbidities and less reserve to handle this early complication and the potential treatments that accompany it such as ablation therapy, repeated cardioversions, RVAD support and/or antiarrhythmic therapies. Finally, VA could simply be a marker of hemodynamic deterioration rather than a cause since the mortality increase continued to occur after the first 30 days defined for early post LVAD VA.

### Limitations

This study is an observational, single center, nonrandomized study. However, most of the findings agree with previously published data from other centers and it has a relatively large sample size with high quality, complete data and includes the most contemporary pumps. This study design cannot determine whether VAs are the cause of the increase in mortality observed, hence does not shed light on whether treating the VAs aggressively would improve survival in this patient population. Multivariate analysis runs the risk of finding a false positive association as a result of multiple models with high number of variables. However, early post LVAD VA remained significant in a multitude of models as we applied our stepwise fashion while avoiding overcorrection of the model. And finally, the mortality analysis for early new onset post LVAD VAs may be underpowered to show a significant result.

## Conclusion

Early post-LVAD VAs are common after LVAD implantation and are associated with a higher mortality in DT patients, especially in the first few months after LVAD implantation. It is also an independent predictor of mortality in this patient population, with most of the mortality difference occurring early during follow-up. Prospective studies are needed to elucidate whether treating these VAs with antiarrhythmic medical or ablation therapy might improve short- and long-term mortality in this patient population.

## Abbreviations

AF: Atrial fibrillation
ATP: Anti-tachycardia pacing
BTT: Bridge to transplant
DT: Destination therapy
ICD: Implantable cardioverter defibrillator
INTERMACS: Interagency Registry for Mechanically Assisted Circulatory Support
LVAD: Left ventricular assist device
LV: Left ventricle/left ventricular
LVEF: Left ventricular ejection fraction
VAs: Ventricular arrhythmias
